# A randomised controlled trial of a community-based healthy lifestyle program for overweight and obese adolescents: the Loozit^® ^study protocol

**DOI:** 10.1186/1471-2458-9-119

**Published:** 2009-04-29

**Authors:** Vanessa A Shrewsbury, Janice O'Connor, Katharine S Steinbeck, Kate Stevenson, Anthea Lee, Andrew J Hill, Michael R Kohn, Smita Shah, Siranda Torvaldsen, Louise A Baur

**Affiliations:** 1 University of Sydney Clinical School, The Children's Hospital at Westmead, Sydney, NSW, Australia; 2 Endocrinology & Adolescent Medicine, Royal Prince Alfred Hospital & University of Sydney, Sydney, NSW, Australia; 3 Academic Unit of Psychiatry & Behavioural Sciences, Institute of Health Sciences, Leeds University School of Medicine, Leeds, UK; 4 Centre for Research into Adolescent's Health, The Children's Hospital at Westmead, Sydney, NSW, Australia; 5 Primary Health Care Education and Research Unit, Sydney West Area Health Service, Sydney, NSW, Australia; 6 Centre for Medical, Psychology and Evidence-Based Decision Making, School of Public Health, University of Sydney, Sydney, NSW, Australia

## Abstract

**Background:**

There is a need to develop sustainable and clinically effective weight management interventions that are suitable for delivery in community settings where the vast majority of overweight and obese adolescents should be treated. This study aims to evaluate the effect of additional therapeutic contact as an adjunct to the Loozit^® ^group program – a community-based, lifestyle intervention for overweight and lower grade obesity in adolescents. The additional therapeutic contact is provided via telephone coaching and either mobile phone Short Message Service or electronic mail, or both.

**Methods and design:**

The study design is a two-arm randomised controlled trial that aims to recruit 168 overweight and obese 13–16 year olds (Body Mass Index z-score 1.0 to 2.5) in Sydney, Australia. Adolescents with secondary causes of obesity or significant medical illness are excluded. Participants are recruited via schools, media coverage, health professionals and several community organisations. Study arm one receives the Loozit^® ^group weight management program (G). Study arm two receives the same Loozit^® ^group weight management program plus additional therapeutic contact (G+ATC). The 'G' intervention consists of two phases. Phase 1 involves seven weekly group sessions held separately for adolescents and their parents. This is followed by phase 2 that involves a further seven group sessions held regularly, for adolescents only, until two years follow-up. Additional therapeutic contact is provided to adolescents in the 'G+ATC' study arm approximately once per fortnight during phase 2 only. Outcome measurements are assessed at 2, 12 and 24 months post-baseline and include: BMI z-score, waist z-score, metabolic profile indicators, physical activity, sedentary behaviour, eating patterns, and psychosocial well-being.

**Discussion:**

The Loozit^® ^study is the first randomised controlled trial of a community-based adolescent weight management intervention to incorporate additional therapeutic contact via a combination of telephone coaching, mobile phone Short Message Service, and electronic mail. If shown to be successful, the Loozit^® ^group weight management program with additional therapeutic contact has the potential to be readily translatable to a range of health care settings.

**Trial registration:**

The protocol for this study is registered with the Australian Clinical Trials Registry (ACTRNO12606000175572).

## Background

### Study context

This study was designed to address the apparent gap in service provision of sustainable community-based weight management programs for overweight and obese adolescents. The Loozit^® ^group weight management program is a modest intensity, evidence-based, healthy lifestyle intervention delivered over a two year period. Instead of providing a higher level of direct contact with participants, this study provides adolescents with additional therapeutic contact via telephone coaching and mobile phone Short Message Service (SMS) and/or electronic mail (e-mail). These modes of communication offer the capacity to provide regular and engaging therapeutic contact with a greater number of adolescents than could be sustained in traditional programs involving only direct contact with participants. Here we give an overview of the literature that has guided the development of this study.

### Obesity in adolescence

The prevalence of adolescent overweight is increasing in almost all developed countries and several developing countries [1] making it one of the most common chronic disorders in this age group [2]. Adolescence is a period of major physical and cognitive change and increasing independence when alterations to eating behaviour, physical activity and other aspects of lifestyle may be maintained into adulthood [3,4]. Adolescence has been termed a "critical period" for the development of adult obesity [5] and adolescents with established overweight or obesity have a moderate to substantial risk of remaining so in adulthood [6]. Overweight and obese adolescents also suffer a range of immediate and long-term medical and psychosocial complications [7,8]. Thus, interventions at this developmental stage may be crucial for present well-being, long-term weight management, and the avoidance of entrenched co-morbidities.

### Obesity management: a continuum of chronic disease care

Obesity is a chronic and often a life-long disease. Therefore its treatment may best be considered within the broad framework of the Chronic Care Model [9] and associated service delivery models, such as the Kaiser model [10]. The Kaiser model proposes that three levels of chronic disease care need to be offered within a health care system. Level 1 care, suitable for the vast majority of affected individuals, emphasises the patient's (or the patient's familiy's) central role in managing their health, in conjunction with primary care doctors and the effective use of community and other health system resources. Level 2 involves care of high risk patients by multidisciplinary disease management protocols, and Level 3 is active case management of highly complex patients [10]. To date, there are no published reports of such a country or state wide approach to obesity management.

### Treatment of adolescent obesity

The conventional components of weight management in adolescents and children include dietary modification, increased physical activity, decreased sedentary activity, behaviour modification and family involvement [11]. There is only limited evidence to guide effective weight management interventions in the adolescent age group that would be sustainable in most health care settings. The 2009 Cochrane review of interventions for treating obesity in children included 27 randomised controlled trials (RCTs) involving adolescents (i.e. mean age > 12 years). The primary type of intervention was behavioural lifestyle modification in 12 studies, pharmacotherapy in 10 studies, physical activity in three studies, and diet in two studies. Meta-analyses indicated a reduction in overweight at 6 and 12 months follow-up in lifestyle interventions with or without the addition of the drugs orlistat or sibutramine [11]. The majority of the interventions included in the 2009 Cochrane review were of relatively high intensity and offered in settings that would be classified as Level 2 or 3 care as per the Kaiser model. There remains a need for more RCTs of adolescent obesity treatments that would be sustainable in Level 1 (i.e. community and primary care) settings.

### Extended therapeutic contact for weight loss maintenance

Sustained weight loss in the long-term (i.e. 2–4 years) following non-surgical treatment of overweight and obesity is modest at best in adults who complete interventions [12] but this outcome may be different in children and adolescents, particularly in those with height growth potential. Few high quality studies have reported long-term outcomes of child and adolescent obesity treatment [11] but in those that have, a high proportion of participants maintained a reduction in overweight up to 10 years after commencing treatment [13,14]. Persisting with positive lifestyle changes appears to be a universal and key factor in successful weight loss maintenance [15,16]. In overweight and obese children and adolescents who have completed an initial course of weight management treatment there appears to be a beneficial effect of extended therapeutic contact (varying from 4–12 months) in slowing weight regain [17,18]. Yet there remains little evidence as to the optimal format and duration that extended therapeutic contact should take for the treatment of adolescent obesity.

### Strategies for extended therapeutic contact for adolescents with chronic disease

The use of telephones [19], mobile phone SMS [20] and interactive technologies such as e-mail or the Internet [21], can be an effective means of providing therapeutic interventions for health behaviour change. These modes of communication are commonly accessible and frequently used by adolescents [22,23] and therefore have the capacity to better engage adolescents in health interventions. Treatments for child and adolescent overweight and obesity have incorporated telephone interventions for over 20 years [11] but the use of e-mail/internet [24-26]and mobile phone SMS [27] in the same role has only emerged in recent years and requires further research in high quality studies [20,21]. Each of these communication modes differs with regards to cost, time required to communicate with multiple recipients, ability to communicate detailed information, and immediacy of access to recipients [28]. Therefore a combination of these communication methods may be an effective means of providing extended therapeutic contact in a weight management program for overweight and obese adolescents.

### Pilot work for a community-based obesity treatment intervention for adolescents

Community health centres in countries such as Australia [29] and the United States [30] are generally located close to residential areas and are therefore a potentially viable setting for adolescent overweight and obesity treatment suitable to Level 1 care as per the Kaiser model. In 2004/2005 we conducted and evaluated a 5-month pilot study of a community based lifestyle intervention for weight management in overweight and obese 13–16 year olds [31]. The pilot intervention consisted of seven group sessions for adolescents and focused upon achievable goals for dietary intake, physical activity, sedentary behaviour, and strategies for increasing self-esteem. The program was named Loozit^® ^by the participating adolescents. Results from that pilot study confirmed the intervention was feasible to run and acceptable to the adolescents. Although the pilot study was statistically underpowered and only of modest intensity, there was a statistically significant improvement in waist circumference, HDL-cholesterol and several of the Harter Self Perception Profile domains (physical appearance and romantic appeal) [31]. Feedback from adolescents and their parents was collected through focus groups and used, together with the group leader's evaluations, to refine the original program's content, structure, and recruitment strategies to form the current Loozit^® ^program. Compared with the original pilot study intervention, the current Loozit^® ^program now includes group sessions for parents/carers and a maintenance phase for adolescents involving 'booster' group sessions and additional therapeutic contact (i.e. via telephone coaching, and mobile phone SMS and/or e-mail) which will be referred to hereafter as ATC. With these modifications the current program is still suitable for Level 1 treatment and meets the recommendations of the Australian *National Health & Medical Research Council Clinical Practice Guidelines *[32].

### Study rationale

To our knowledge, the current study is the first RCT of a sustainable, community-based, weight management program for overweight and obese adolescents that will evaluate the effect of ATC, in the form of a combination of communication strategies (i.e. via telephone coaching, and mobile phone SMS and/or e-mail) as an adjunct to a group program over a two year period.

## Aims and hypotheses

The primary aim of the study is to determine the effect of ATC to usual treatment on body mass index (BMI) z-score and waist circumference z-score in overweight and obese adolescents aged 13–16 years (at baseline) who participate in a community-based weight management program (the Loozit^® ^group program). Adolescents enrolled in the study are randomised to receive one of the following two interventions: the Loozit^® ^group program (G); or the Loozit^® ^group program + additional therapeutic contact (G + ATC). ATC includes a combination of telephone coaching, and mobile phone SMS and/or e-mail messages. It is hypothesised that:

1. compared to baseline, both the G and the G+ATC interventions will result in a clinically relevant and a statistically significant:

a) reduction in BMI z-score and in waist circumference z-score (primary outcomes) at 2, 12 and 24 months post-baseline;

b) improvement in other important outcome measures in weight management including metabolic profile indicators, eating patterns, physical activity, sedentary behaviour, and psychosocial well-being (secondary outcomes) at 2, 12 and 24 months post-baseline; but that

2. the G+ATC intervention, relative to the G intervention, will produce a clinically relevant and a statistically significant greater reduction in the primary and secondary outcomes outlined above at 12 and 24 months post-baseline, as well as better retention in the program.

## Methods and design

### Study design

**Figure 1:**
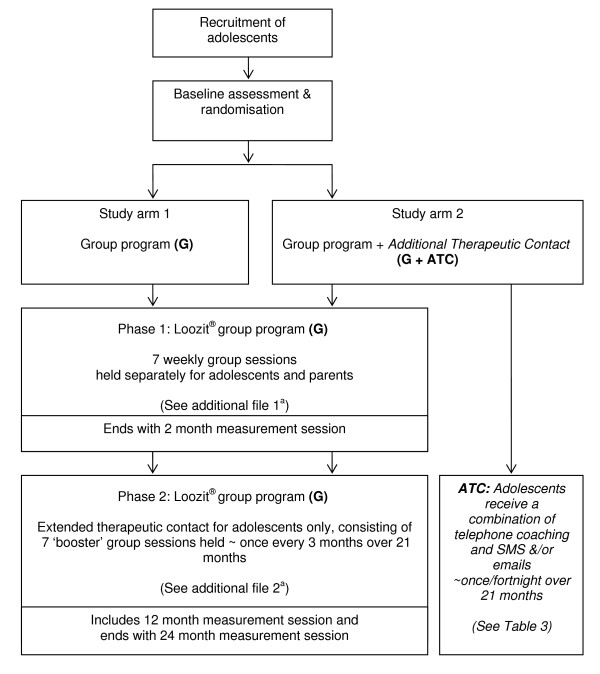
**Study design**. ^a ^See additional file 1: Phase 1 of the Loozit^® ^group program – weekly topics and key content. ^b ^See additional file 2: Phase 2 of the Loozit^® ^group program – 'booster' session topics and key content

The overall study design is summarised in Figure 1. This study is registered with the Australian Clinical Trials Registry (ACTRNO12606000175572) and has been approved by the Human Research Ethics Committees of: The Children's Hospital at Westmead, Sydney West Area Health Service, and The University of Sydney.

### Participants

#### Recruitment

Adolescents are being recruited in Sydney, Australia, via the media, schools, health professionals and several community organisations. The Children's Hospital at Westmead Public Relations department has contacted various media outlets and subsequently articles about the study have appeared in local and city-wide newspapers and, to a lesser extent, aired on television and radio programs. Local schools are approached with regards to placing information about the study in school newsletters, and school counselors are provided with information about the study. For some cohorts the study research assistants presented information at school meetings attended by staff, students, or parents and citizens. Health professionals working within the local area are informed about the study through faxes, flyers, e-mail bulletins and, less frequently, presentations are given. Other recruitment strategies include: placing flyers on notice boards in youth centers, local gyms and pharmacies; contacting local weight management businesses; contacting large businesses within the local area and asking them to include study information in their staff newsletters; and participants recommending the study to friends. For one cohort, flyers were distributed to households via a letter box drop in the local area of the community health centre at which the study was to be conducted.

#### Eligibility criteria

The inclusion and exclusion criteria for assessing adolescent eligibility to participate in the study (Table 1) are used during a telephone screen that is conducted with whoever enquires about the study (e.g. parents/carers, adolescents, health professionals or school counselors). Each enquiry about study participation is issued with a unique identification number that is used to distinguish individuals throughout the study. Adolescents who meet the study criteria are invited to an appointment with a research assistant, along with their parents/carers, in order to confirm eligibility based on objectively measured weight status. Informed consent or assent, depending on age, is obtained from adolescents who meet the study eligibility criteria and wish to participate. Consent is also obtained from one of the adolescent's parents/guardians. Although many adolescents are found to be ineligible during the telephone screen, it is uncommon for adolescents to be found ineligible at the appointment. Adolescents who are ineligible to participate, and their parents/carers, are encouraged to follow up with their general practitioner.

**Table 1:** Inclusion and exclusion criteria for adolescent participation in the Loozit^® ^study

**Inclusion criteria**	**Exclusion criteria**
▪ Age 13.0–16.9 years	▪ Severely obese (i.e. BMI z-score^a ^>2.5) or if there is a secondary cause for overweight/obesity
▪ Overweight to moderately obese (i.e. BMI z-score^a ^range 1.0–2.5)	▪ Intellectual disability, significant medical illness, psychiatric disturbance
▪ Home access to a landline telephone	▪ Taking medications that affect weight status
▪ Home access to the internet to receive e-mails or access to a mobile phone to receive SMS messages	▪ Inability to take part in physical activity sessions
▪ Ability to attend the group program for 7 weeks in the first instance on the specified days^b^	▪ Poor level of spoken English (adolescent or parent/carer)
▪ At least one of the adolescent's parents/carers must be willing to also participate in the initial 7 parent group sessions	

^a ^calculated using Centers for Disease Control growth reference [41]

^b ^The 'G' and 'G +ATC' group programs are run on separate weeknights and therefore potential participants need to be available to attend on either evening, pending randomisation, even though they would only attend once per week

#### Randomisation

A computer generated randomisation sequence, stratified for sex, age group (13–14 years; 15–16 years), and intervention site was provided by staff in the Clinical Epidemiology Unit, The Children's Hospital at Westmead. The trial manager is responsible for concealed allocation which involves preparing, for each intervention site, sex and age group, a set of consecutively numbered opaque envelopes containing the group allocation 'G' or 'G+ATC'. After participant consent is obtained the next numbered envelope is opened by a research assistant (at the abovementioned appointment to confirm eligibility), revealing the group allocation and this is recorded along with the individuals pre-assigned identification number. Outcome assessors are the only persons blinded to group assignment.

#### Sample size

In adults, the clinical benefit of 5% relative weight loss is associated with improvements in HbA1c in Type 2 diabetes [33], blood pressure [34], total cholesterol and HDL cholesterol [35], and ovarian function in polycystic ovarian syndrome [36]. It could be reasonably assumed that adolescents will experience similar clinical benefits. Over a one year period, a clinically significant weight loss of 5% corresponds to a 0.4 unit change in BMI z-score, and a 5% reduction in waist circumference corresponds to a 0.2 unit reduction in waist circumference z-score. A sample size of 64 in each intervention arm will have 80% power to detect a 0.4 unit difference in mean change of BMI z-score from baseline to 2, 12 and 24 months follow-up in the two interventions (two group t-test, 0.05 two-tailed significance). Based on the results from our preliminary study, we have assumed the common SD is 0.8 for the change in BMI z-score, and that the calculated sample size is sufficient to detect a 0.2 unit change in waist circumference z-score, an 8% increase in HDL cholesterol, a 9% decrease in total cholesterol, a 7 mmHg and a 4 mmHg reduction in systolic and diastolic blood pressure, respectively, and a 15% increase in Harter global self-worth score [31]. To accommodate a 30% drop-out rate (based on adult studies), we aim to enrol 84 participants in each intervention arm.

### Study interventions

The specific details of the interventions received by study arms: 1) Loozit^® ^group program 'G' only; and, 2) Loozit^® ^group program 'G' plus additional therapeutic contact 'ATC' i.e. 'G+ATC', are as follows:

#### 1) Loozit^® ^group program (G)

##### i) Background

The key aspects covered in the Loozit^® ^group program are in keeping with Australian clinical guidelines for the management of overweight and obesity [32] and include healthy food choices and eating patterns, increasing physical activity and reducing sedentary behaviour, strategies for managing behaviour change with a focus on goal setting, dealing with stress and building self-esteem. The program is based upon a cognitive behavioural approach using behavioural principles to change dietary intake and activity levels, and social cognitive approaches to modify self-efficacy, motivation, perseverance and self-regulation [37].

Participants in both arms of this study receive the Loozit^® ^group program (G) which is provided separately to adolescents and their parents/carers. Separate skills training sessions for parents have been shown to help weight loss in adolescents [38], and this approach was requested by both parents and adolescents in focus groups held as part of the pilot study that preceded the current study [31]. The Loozit^® ^group program is run over a 24 month period and is divided into two distinct phases. Phase 1 consists of seven group sessions for adolescents and their parents/carers that take place weekly for seven consecutive weeks during a school term. This is followed by phase 2 which involves a further seven group sessions for adolescents, called 'booster' group sessions, held once every school term (approximately once every three months to 24 months post-baseline).

##### ii) Phase 1 – Adolescent and parent weekly group sessions

###### a) Format

Separate but simultaneous group sessions are held for adolescents and their parents/carers in the evening. Group sessions are facilitated by dietitians (but could be run by nurses or other health professionals) and each group typically has between five and nine members. Usually one parent/carer from each family attends but there are no restrictions on parent/carer numbers. Parents/carers are not permitted to bring younger children to the group sessions in order to minimise disruption. Due to budgetary constraints we are unable to provide child minding facilities for adolescent's siblings. The general structure of an adolescent and parent group session is outlined in Table 2.

**Table 2:** Structure of weekly group sessions in phase 1 of the Loozit^® ^group program

**Adolescent session**	**Time** **(mins)**	**Parent session**	**Time** **(mins)**
i) Reconnect with group (includes an icebreaker) and review progress with goals	15	i) Reconnect with group and review progress with family goals	15
ii) Resistance activities	10	ii) Facilitator-led discussion on set topic^a^	50
iii) Facilitator-led discussion on set topic^a^	30	iii) Recap important points and record family goals for the week ahead	10
iv) Recap important points and record individual goals for the week ahead	10		
v) Fun active game and light refreshments	10		
Total	75	Total	75

^a ^See additional file 1: Phase 1 of the Loozit^® ^group program – weekly topics and key content

###### b) Content

For a summary of the topics and key content of the facilitator-led discussion in each weekly session (see additional file 1: *Phase 1 of the Loozit^® ^group program – weekly topics and key content*). The group sessions are designed to provide education, to promote adolescent skill development, to encourage group interaction, and to foster peer bonding. The main difference between the structure and content of the adolescent and parent group sessions is that those for adolescents promote physical activity skills development through approximately 20 minutes/session of indoor resistance activities and fun active indoor games. Adolescents also spend more time discussing self-esteem and stress management and have a practical session involving the preparation and tasting of healthy foods. The parent sessions focus on practical support of behavioural change in adolescents and parental role modeling of healthy lifestyle changes for the family unit. By the end of phase 1 adolescents and parents/carers have largely covered the same material.

###### c) Goal setting

In the initial session, adolescents and parents/carers are taught how to set "SMART" goals that are ***s***pecific, ***m***easurable, ***a***chievable, ***r***ealistic, and can be attained in a fixed ***t***ime frame. Towards the end of each group session, adolescents are given time to set an individual SMART goal for the week ahead that relates to the current or previous session. Adolescents record their goal on a brightly coloured, take home, 'UZIT' worksheet. Similarly, at the end of each group session parents are given time to set 'family goals' that are recorded in the parent booklet; an example of such a goal would be to have three family meals at the table with the television turned off that week. At the beginning of the adolescent and parent group sessions, facilitators encourage group members to report to the group on their progress with their goals and individual strategies in order to support and educate each other. Success and effort is praised, and facilitators encourage group discussion on solutions to overcoming the barriers to goal achievement.

###### d) Resources

The Loozit^® ^group program is delivered using a limited array of inexpensive resources in line with the concept of sustainability. Separate, specially developed booklets for adolescents and parents/carers are used during the sessions to support learning and include a summary of the key information discussed, along with space for occasional written activities and recording goals. Parents/carers are given their booklets to take home each week, whereas the adolescents' booklets are collected at the end of each session by the group facilitator to ensure they will be available at each session. At the end of phase 1, adolescents are given their booklets to keep at home. The resistance-based physical activities do not require equipment other than tinned food cans which are used as inexpensive hand weights. Basic equipment is used for a variety of fun active games including skipping ropes, rubber balls, and balloons. Adolescents are also encouraged to introduce their own fun games and bring along their favourite music to play during the game time. The resources used for the adolescent cooking activity include common kitchen utensils, serving plates, and the venue's microwave. Selected food models and food packages are used for educational demonstrations. Light refreshments are provided at each group session.

##### iii) Phase 2 – Extended therapeutic contact (adolescents only)

###### a) Format and content

At the completion of phase 1, adolescents in both study arms enter phase 2 which involves extended therapeutic contact and forms the maintenance component of the Loozit^® ^group program 'G'. Phase 2 involves seven group sessions, held approximately once every three months in the middle of the school term (four terms per year), comprising five × 60 minute 'booster' group sessions and an outcome assessment session at 12 and 24 months post-baseline (see additional file 2: *Phase 2 of the Loozit^® ^group program – 'booster' session topics and key content*). The main educational content of the booster group sessions is generally new, although the key messages from phase 1 are reinforced (see additional file 1: *Phase 1 of the Loozit^® ^group program – weekly topics and key content*). Booster group sessions have a structure similar to the group sessions held in phase 1 (Table 2); however the amount of time spent on various components is less. For example, more time is spent on the fun group building activity and less time is spent on the facilitator-led discussion. No extended therapeutic contact is offered to parents/carers.

###### b) Practical considerations

In order to preserve 'booster' group numbers when attrition occurrs, it was decided that groups allocated to the same study arm, from various cohorts that attended the same venue, should be combined. In order to prevent contamination between the two study arms, participants in the 'G' and 'G+ATC' interventions are never put together in the same group. This combining of groups in the maintenance phase also permits an informal peer support system where participants in the latter stages of the program provide an example and encouragement for participants in the early stages of phase 2.

#### 2) Additional therapeutic contact for adolescents (ATC)

##### i) Background

In addition to the Loozit^® ^group program 'G', participants in the 'G+ATC' study arm also receive a modest level of additional therapeutic contact (ATC) in phase 2 only. ATC is planned communication from the group facilitator to the adolescent via telephone coaching plus a mode of electronic communication i.e. mobile phone SMS or e-mail messages, or both, according to adolescent preference. The aim of ATC is to enhance adolescents' knowledge, skills and confidence to initiate and maintain required changes in dietary and activity behaviours. Because mobile phone SMS and e-mail interventions are relatively new technologies, there is little evidence in the child and adolescent weight management literature on the optimum frequency of contact and level of interaction in using these technologies, especially in the context of a program that already offers extended therapeutic contact in the form of group sessions and telephone coaching. A primary consideration in the design of the ATC schedule was that it was in keeping with the sustainability aspects of the Loozit^® ^group program. Parents/carers of adolescents in the 'G+ATC' study arm do not receive any form of ATC.

##### ii) Format

The schedule for ATC covers a 10 week school term period and the following holiday period (Table 3). Throughout the study the schedule is repeated seven times, totaling 32 electronic communication messages and potentially 14 telephone coaching calls over approximately 21 months.

**Table 3:** Quarterly schedule for additional therapeutic contact (ATC) in the 'G+ATC' intervention group^a^

**Quarterly schedule**	**Mode of ATC**
***School term week***	
1, 4, 6, 9	No contact
2 & 7	10 minute telephone coaching call with adolescent
3, 8,10	Group facilitator sends adolescent an email message and/or mobile phone SMS according to preference
5	Booster group session

***End of term school holiday week*^b^**	
1	No contact
2	Group facilitator sends adolescent an email message and/or mobile phone SMS according to preference

^a ^This schedule is repeated seven times during phase 2 of the study

^b ^Once a year students have a six week summer holiday. During this period they receive ATC once a fortnight via mobile phone SMS and/or e-mail, totaling 3 contact occasions.

##### iii) Telephone coaching

Telephone coaching is formalised communication between the adolescent and the group facilitator and is adapted from that used in a study involving adolescents with type 1 diabetes [39]. Semi-structured telephone coaching calls are designed to be approximately 10 minutes in duration with elements to: a) establish rapport; b) collaborate to identify behavioural goals related to topics covered in the group program (or previously identified by the adolescent); c) identify barriers to achieving goals; d) assist in problem solving; and e) provide positive encouragement for further change [39]. If the adolescent is not contactable, facilitators continue to attempt to contact the adolescent in line with the developed protocol.

##### iv) Mobile phone SMS and e-mail messages

Mobile phone SMS and e-mail communications are researcher-initiated short messages (less than 160 characters) that cover the important points raised during phase 1. The messages follow a set schedule so that all participants receive the same messages. To encourage interactivity approximately two-thirds of the messages end with 'please reply' for example, *"Everywhere is within walking distance if you have the time" How could you be more active this week? Please reply"*. If a response is received from an adolescent, the facilitator is permitted one reply message that is individually tailored with the purpose of providing positive reinforcement, education and encouragement.

##### v) Protocols

Detailed written protocols are followed for the semi-structured telephone coaching and the pre-determined content of the mobile phone SMS and e-mail messages. These were developed based upon those used in studies with adults and adolescents with type 1 diabetes [39,40]. Facilitators keep a detailed record of ATC implementation including delivery medium, time/duration, and content of every contact initiated with adolescents. The same details are recorded for all responses from adolescents. Strict protocols have been developed to guide facilitators in the event of a child protection, mental health or other major issue being raised during ATC.

##### vi) Adolescent ATC preferences

Adolescents' ATC preferences regarding the day/s and time/s that they wish to receive telephone coaching calls, as well as their preference to receive mobile phone SMS and/or e-mail messages is determined at the two month outcome assessment session at the end of phase 1. ATC preferences are reassessed at the 12 month outcome assessment session. Adolescent contact details relevant to ATC are collected at the end of phase 1 and these are checked on a regular basis i.e. during the telephone coaching sessions and at the 12 month outcome assessment session.

### Intervention sites

Community Health Centres or local government community centres at a range of sites in Sydney, Australia, have been approached with regards to the potential of using group rooms for phase 1 and 2 of the Loozit^® ^group program over two years. Each venue requires a room large enough to use for indoor active games and resistance exercise activities, adequate parking, and access to a microwave and sink for the adolescent cooking session. Although proximity to public transport is a consideration, the vast majority of families travel to the groups by private vehicle. Many of the venues have free parking. When there is a parking charge participants are given a voucher to cover expenses.

### Quality control

#### Staff training

Group facilitators (and outcome assessors) receive standardised training. A facilitators' manual contains detailed information specific to each group session including a script for the educational material, timing of each component, the type of resistance activities to be performed, suggestions for various fun active games, and a list of the resources required. Group facilitators attend a weekly meeting with the project manager to ensure protocol adherence.

#### Evaluation of group sessions

Facilitators complete a standardised evaluation form after each group session to record parent/carer and adolescent attendance at group sessions and the degree of compliance with the facilitator's manual during the group session.

### Measures

#### Schedule

The primary outcomes are adolescent BMI z-score and waist circumference z-score. The secondary outcomes are metabolic profile indicators, physical activity, sedentary behaviour, food intake, eating patterns, and various aspects of adolescent psychosocial wellbeing. All these outcomes are measured at baseline, 2 months post-baseline (end of phase 1), 12 months post-baseline (almost half-way through phase 2), and 24 months post-baseline (at the end of phase 2). In addition, at 2, 12 and 24 months post-baseline, adolescent and parent/carer satisfaction with the program is measured by questionnaire.

#### Measurement tools and procedures

##### i) Anthropometry and blood pressure

Trained staff members, who are blinded to treatment allocation, measure adolescents' anthropometry and blood pressure (BP) using standard procedures and calibrated instruments. Weight is measured with portable scales (Tanita HD-316, Tanita Corp.;Tokyo, Japan) to the nearest 0.1 kg, with shoes and heavy clothing removed. Height is measured to the nearest 0.1 cm using a fixed stadiometer at The Children's Hospital at Westmead (Holtain Limited; Crymych, Dyfed, Britain) or a portable stadiometer (Seca, Model 220; Germany) when measurements are conducted at Community Health Centres. Waist circumference is measured at the narrowest point between the lower costal (rib) border and the iliac crest using a nonextensible steel tape. BMI and waist circumference z-scores will be calculated based upon age- and sex-specific reference values [41,42]. Systolic and diastolic BP are measured using an automated BP monitor (Dinamap model 8101, Critikon Inc.; Florida, USA) under standard conditions [43]. All of these measurements take place in a private room.

##### ii) Metabolic bio-markers

A 20 ml fasting blood sample is collected by a nationally accredited external pathology laboratory (also blinded to the adolescent's treatment allocation) which has many collection sites in the study region. The blood sample is analysed for cholesterol (total, HDL & LDL), triglycerides, insulin, glucose and liver function indicators using standard automated techniques in that laboratory.

##### iii) Adolescent self completed questionnaires

The following questionnaires are completed by adolescent participants, in a room with other members of their group, supervised by a group facilitator. Together these questionnaires take between 30–45 minutes to complete.

###### a) Adolescent physical activity and sedentary behaviour

The Children's Leisure Activities Study Survey is used to assess physical activity and sedentary behaviours [44]. This has been developed for the Australian environment and is validated in 10–12 year olds.

###### b) Adolescent food intake and eating patterns

This questionnaire includes 15 food frequency questions on food and beverage items relevant to obesity [45] as well as questions on eating patterns and behaviours that were developed for an Australian study of adolescents' eating patterns and behaviours [46].

###### c) Adolescent psychosocial wellbeing

Several measures of adolescent psychosocial wellbeing are used including:

▪ The Harter Self-Perception Profile for Adolescents [47] which provides a measure of global self worth and perceived competence in eight specific domains, such as social acceptance, athletic competence and, physical appearance.

▪ Adolescents' perception of body image, including present and preferred body shape, is assessed using a simple visual scale that has been validated [48].

▪ The MacArthur Scale of Subjective Social Status, an adaptation of a 10-point vertical ladder scale, is used to evaluate social acceptance with adolescent peers [49].

▪ The Mental Health Inventory-5 (MHI-5), a 5 question mental health assessment component of the SF-36, is used to assess quality of life for adolescents [50].

###### d) Pubertal stage

Adolescents self-report their stage of pubertal maturation using the standard Tanner Stage line drawings for males or females [51] at baseline, and 12 and 24 months post-baseline.

###### e) Adolescent satisfaction with the program

At 2, 12, and 24 months post-baseline, all adolescents complete a questionnaire, adapted from one used by the investigators in another study involving obese pre-adolescent children [52], addressing the quality of service provided and whether participants would recommend the program to others. A separate questionnaire is used at 24 months post-baseline in the 'G+ATC' group which evaluates the types of ATC communication (i.e. telephone coaching, mobile phone SMS, e-mails) that adolescents found to be most useful.

##### iv) Parent/carer self completed questionnaires

At baseline parents/carers complete a short questionnaire regarding demographic characteristics. Parents/carers also complete a questionnaire to assess their satisfaction with the study interventions at 2, 12, and 24 months post-baseline, similar to the one completed by adolescents.

### Planned data analysis

Variables that are not normally distributed will be transformed to make them so, or non-parametric techniques will be used. Analysis of covariance will be used to test for differences in baseline characteristics by study arm and adjustment will be made to analyses as required. An estimation of intervention effect on outcome measures will be obtained at each follow-up observation (2, 12, & 24 months post-baseline) on an intention to treat basis. A mixed model will be used to test the average effect of the intervention after taking account of baseline covariates. A time-group interaction will be included to test whether the rate of change of BMI z-score is different between the 'G' and 'G+ATC' study arms. Planned contrasts will be used to measure between group differences at each evaluation time point. Randomisation should ensure distribution of ethnicity but ethnicity subgroup analysis and statistical adjustment will be made as required.

### Time plan for the Loozit^® ^RCT

Recruitment began in May 2006 and by July 2011 it is anticipated that all participants will have completed 24 month outcome assessments.

## Discussion

This study is the first RCT of adolescent overweight and obesity management to use a combination of methods to provide ATC as an adjunct to a healthy lifestyle program which is potentially sustainable to run in community settings. The results of this RCT will determine if ATC improves treatment outcomes in overweight and obese adolescents participating in the Loozit^® ^group program. This study also has the capacity to provide useful information regarding the practical aspects of running an adolescent weight management intervention in a community setting, and providing ATC to adolescents via a range of communication modes. If shown to be successful, the Loozit^® ^group weight management program with ATC has the potential to be readily translatable to a range of health care settings.

## Competing interests

The authors declare that they have no competing interests.

## Authors' contributions

The study chief investigators JO, LAB, KSS, AJH, MRK and SS were responsible for identifying the research question, design of the study, obtaining ethics approval, the acquisition of funding, and overseeing study implementation. JO, AL, KS, LAB, AJH and VAS have contributed to developing the precise content of the study interventions and resources, and/or recruiting participants, and/or study implementation. JO, LAB, KSS, ST and VAS have been involved in developing a detailed analysis plan for the study. All authors were responsible for the drafting of this manuscript and have read and approved the final version.

## Pre-publication history

The pre-publication history for this paper can be accessed here:



## Supplementary Material

Additional file 1 **Phase 1 of the Loozit^® ^group program: weekly topics and key content**. Topics and content covered in the parent and adolescent group sessions. Includes guidelines promoted in both adolescent and parent sessions. Click here for file

Additional file 2 **Phase 2 of the Loozit^® ^group program: 'booster' session topics and key content**. Topics and content covered in the adolescent 'booster' group sessions. Click here for file
