# Supplementary material for: A randomised controlled trial of a community-based healthy lifestyle program for overweight and obese adolescents: the Loozit® study protocol
Source: BMC Public Health. 2009 Apr 29;9:119. doi: 10.1186/1471-2458-9-119 (PMC2687446; doi:10.1186/1471-2458-9-119)
Supplement: Additional file 1 — Phase 1 of the Loozit® group program: weekly topics and key content. Topics and content covered in the parent and adolescent group sessions. Includes guidelines promoted in both adolescent and parent sessions. [file 1471-2458-9-119-S1.doc]

**Additional file 1: Phase 1 of the Loozit® group program -** weekly topics and key content

| **Week** | **Adolescent sessions** | **Parent sessions** | **Guidelines promoted in both adolescent and parent sessions** |
| --- | --- | --- | --- |
| **1** | ***Benefits of a Loozit® healthy active lifestyle***   - Discuss participants’ expectations and motivation - Establish group goals and rules - Energy balance and successful weight management - Steps to kick start a healthy active lifestyle - Learn how to set ‘SMART’ healthy lifestyle goals | ***Introduction to Loozit®; Importance of supporting your adolescent; Goal setting***   - Discuss participants’ expectations and motivation - Establish group goals and rules - Factors that influence adolescent body weight and successful weight management - A family approach to adolescent weight management and the role of parents in establishing a supportive home environment - Steps to kick start a healthy active lifestyle for the family - Learn how to set ‘SMART’ family-focused goals | Steps to kick start a healthy active lifestyle:   - enjoy breakfast each day - eat balanced meals - each day aim for 1 hour of physical activity and less than 2 hours screen time - choose water as the main drink - keep fat intake low - increase vegetables - goal setting |
| **2** | ***Increasing physical activity; Reducing sedentary behaviour***   - Identify benefits of physical activity - Exercise intensity and resistance activites - Organised and incidental activity - Strategies for becoming more active and reducing screen time | ***Increasing physical activity; Reducing sedentary behaviour***   - Identify benefits of physical activity and strategies for encouraging adolescents and the whole family to be more active and reduce screen time - Role modeling physical activity - Positive reinforcement of adolescents efforts to be active - Common barriers to being more active and ways to overcome them | Each day aim for:   - at least 1 hour of moderate intensity activity - less than 2 hours of ‘screen time’ - 8 to 9 hours of sleep each night |
| **3** | ***Healthy eating for adolescents***   - Macronutrients – carbohydrates, protein and fat - Overview of the core food groups^a^ (‘everyday foods’), the better choices within each group and, recommended servings and portion sizes - Identification of non-core foods (‘sometimes foods’) and strategies to limit intake - Strategies to reduce fat intake and increase fibre intake - Strategies to increase water intake and reduce high-sugar fluid consumption - Balanced eating for adolescents and meal ideas for healthy breakfasts and dinners - Strategies for increasing vegetable intake | ***Healthy eating for your adolescent and your family***   - Macronutrients – carbohydrates, protein and fat - Overview of the core food groups^a^ (‘everyday foods’), the better choices within each group and, recommended servings and portion sizes - Identification of non-core foods (‘sometimes foods’) and strategies to limit intake - Strategies to reduce fat intake and increase fibre intake - Strategies to increase water intake and reduce high-sugar fluid consumption | - Enjoy 3 balanced meals and 2-3 nutritious snacks each day - Ensure each meal includes carbohydrates & protein, is low fat & high in fibre - Aim for the recommended serves per day from each of the food groups - Choose water as the main drink: aim for 6-8 glasses/day - Increase your vegetable intake - Choose low fat dairy foods |
| **4** | ***Food labels; Measuring fat and sugar in food and drinks; Lunch box and snack ideas***   - Practical session on reading nutrition information panels and ingredients lists on food products (focus on fat, fibre and sugar) - Practical exercise: measuring the amount of fat and sugar in various food and drinks - Easy lunch box and snack ideas | ***Shopping; Food labels; Fat and sugar in foods and drinks; Balanced meal ideas***   - Practical session on reading nutrition information panels and ingredients lists on food products (focus on fat, fibre and sugar) - Visual presentation of the amount of fat and sugar in various food and drinks - Balanced eating for adolescents and meal ideas for healthy breakfasts, lunch boxes, afternoon snacks, and dinners - Role modeling healthy balanced eating - Tips for grocery shopping | - Enjoy breakfast each day - Increase fibre intake - Pack a nutritious lunch box (include a lunch meal, 1 piece of fruit, 1 healthy snack and a water bottle) - Aim for less than 400kJ per snack - Enjoy up to 3 serves of ‘extras’ per week |
| **5** | ***Positive self esteem***   - Overview of the importance of a healthy self-esteem and influencing factors - Individually adolescents write down their personal strengths in various domains of their lives - Strategies for positive thinking, self-talk, and building self-esteem - Practical exercise: blinded taste testing of regular and reduced kilojoule food and drink products | ***Recipe modification; Family food habits; Parent / Carer role in building self esteem***   - Strategies for modifying recipes to reduce fat and sugar content, and increase fibre content - Identify family food habits that hinder healthy eating and positive ways to modify the family food environment - Families should aim to eat at least one meal every day together at the dining table without the TV - Overview of self-esteem and strategies for building a healthy self esteem in adolescents - Strategies for minimising stress in adolescents in ways that support a healthy lifestyle | - Think positive, talk positive |
| **6** | ***Stress management***   - Overview of stress and personal stress indicators - Strategies to relieve stress in positive ways that support the Loozit**®** healthy active lifestyle | ***Healthy take away options; Eating out; Gatherings with family and friends***     - Advised to limit high fat, high kilojoule takeaway to once per fortnight - If eating takeaway foods more than once a fortnight, aim for less than 2000kJ per meal - ‘Better options’ for take away foods and when eating out - Strategies for enjoying frequent special occasions (where food is involved) that supports weight management - Healthy celebration foods | N/A |
| **7** | ***Review and maintenance***   - Review important points from sessions 1-6 - Recognition of changes and progress-to-date - Apply knowledge by completing a case study as group - Steps for overcoming barriers to achieving and maintaining a healthy lifestyle - Preparation and tasting of nutritious snacks (wholemeal mini-pizzas, fruit kebabs, vegetable sticks with low fat dip) | ***Review and maintenance***   - Review important points from sessions 1-6 - Recognition of changes and progress-to-date - Steps for overcoming barriers to achieving and maintaining a healthy lifestyle |  |

^a^ As per the Australian guide to healthy eating [53]
