# Supplementary material for: A randomised controlled trial of a community-based healthy lifestyle program for overweight and obese adolescents: the Loozit® study protocol
Source: BMC Public Health. 2009 Apr 29;9:119. doi: 10.1186/1471-2458-9-119 (PMC2687446; doi:10.1186/1471-2458-9-119)
Supplement: Additional file 2 — Phase 2 of the Loozit® group program: 'booster' session topics and key content. Topics and content covered in the adolescent 'booster' group sessions. [file 1471-2458-9-119-S2.doc]

**Additional file 2: Phase 2 of the Loozit® group program - ‘booster’ session topics and key content**

| **Session** | **Topics and key content** |
| --- | --- |
| 1 | ***Healthy take away food options; Eating out; Special occasions***  1.  This session aims to help adolescents to identify the better take away food options in terms of fibre, fat, and total energy (kJ)  2.   Adolescents are encouraged to limit high fat, high kilojoule take away food to once per fortnight  3.   Adolescents complete a practical activity that involves calculating total energy (kJ) and fat content (g) in their usual take away meal and snacks choices using nutrition information tables downloaded from websites. As a general rule adolescents are encouraged to choose meals with less than 10g fat/100g and less than 2,000kJ. Healthier alternate choices and modifications to existing ‘meal deals’ are discussed |
| 2 | ***Increasing physical activity – overcoming the barriers***   1. Re-cap physical activity and screen time guidelines and benefits of physical activity 2. Strategies for including more incidental activity every day and reducing screen time 3. Strategies for overcoming barriers to increasing physical activity |
| 3 | **12 month measurement session** |
| 4 | ***Portion sizes; Eating cues; Overcoming non-hungry eating***   1. Recognising hunger and fullness 2. Revisit portion sizes for different food groups 3. Overcoming non-hungry (emotional eating) and overeating |
| 5 | ***The ins & outs of fad diets and reality TV weight loss programs***   1. Characteristics of fad diets versus healthy weight loss programs 2. Discussion about reality TV weight loss programs – resources, safety, long term outcomes, applicability to real-life 3. Review Loozit, Healthy Active Living, key points |
| 6 | ***Time Management – making the most of your day***   1. Identify individual priorities and time wasting activities 2. Discuss time management strategies 3. Work through time management case study as a group |
| 7 | **24 month measurement session** |
